# Supplementary material for: Capturing effects of blood flow on the transplanted decellularized nephron with intravital microscopy
Source: Sci Rep. 2023 Mar 31;13:5289. doi: 10.1038/s41598-023-31747-w (PMC10066218; doi:10.1038/s41598-023-31747-w)
Supplement: Supplementary file 1 — Supplementary Figure 1. [file 41598_2023_31747_MOESM1_ESM.pdf]

Title: A comparison of the disruptions to scaffold integrity observed after autologous and allogenic scaffold transplantation during the week after implantation.

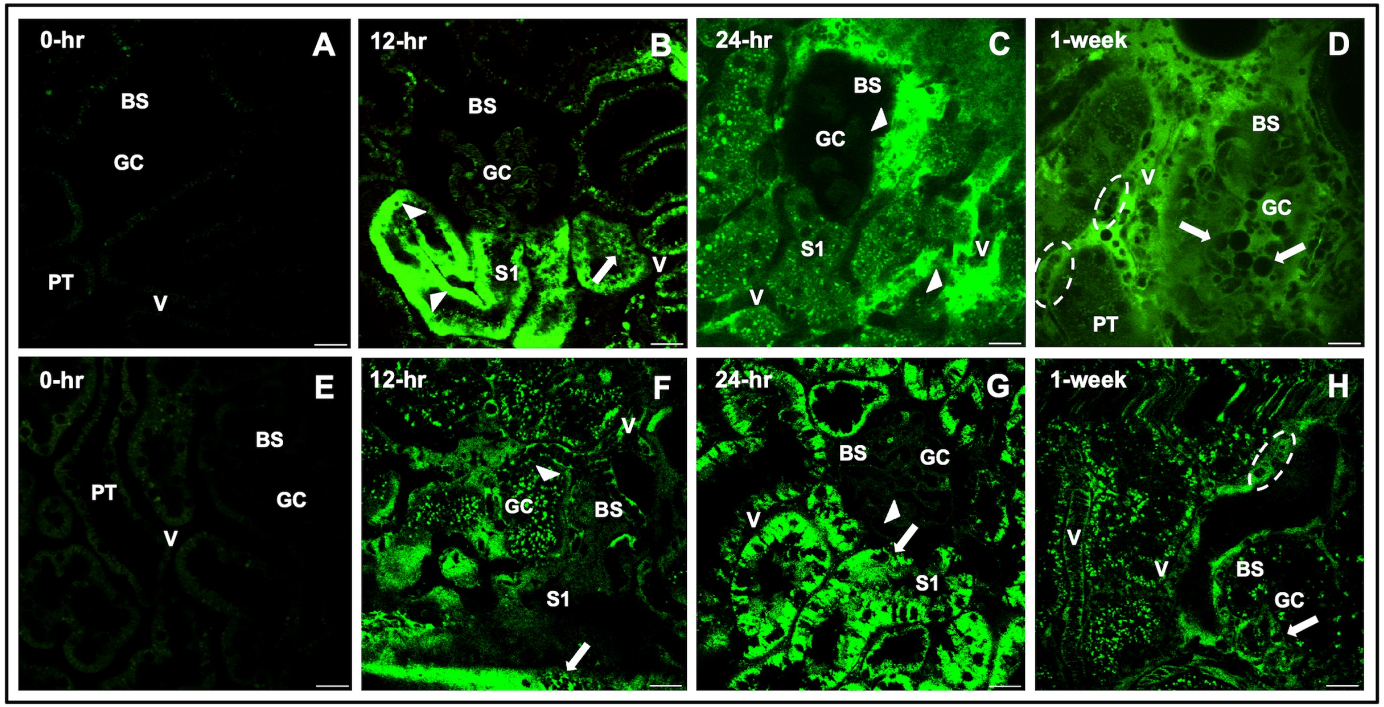

Supplemental Figure 1: Disruptions to scaffold integrity during a week after transplantation illustrating alterations to the decellularized glomerular and tubular and vascular compartments observed in autologous (images A through D) and allogeneic (images E through H) transplantation recipients. These images were taken at various time points across the 1-week measurement period: images A and E were taken at the 0-hour time point; images B and F were taken at the 12-hour time point; images C and G were taken at the 24-hour time point; and Images D and H were taken at the 1-week time point. The arrowheads highlight regions where substantial and inhomogeneous levels of dye translocation into luminal, epithelial, and interstitial compartments can be observed. Arrows highlight the presence of bleb/vesicle formation that accompanied dye extrusion from breached decellularized glomerular. Dashed ovals are used to identify rouleaux formation within the decellularized vascular tracks.
